# The interferon-induced MxB protein inhibits an early step of HIV-1 infection

**DOI:** 10.1186/1742-4690-10-S1-P48

**Published:** 2013-09-19

**Authors:** Zhenlong Liu, Qinghua Pan, Shilei Ding, Jin Qian, Fengwen Xu, Jinming Zhou, Shan Cen, Fei Guo, Chen Liang

**Affiliations:** 1 Lady Davis Institute, Jewish General Hospital, Montreal, Quebec, Canada; 2 Institute of Pathogen Biology, Beijing, China; 3 Institute of Medicinal Biotechnology, Beijing, China

## Background

Interferon protects cells from virus infection by inducing the expression of genes with antiviral activities. One such antiviral gene is Mx (myxovirus resistance) that was first identified in mice for its protection against influenza virus infection. Humans carry two Mx genes, MxA and MxB. Although MxA has been reported to inhibit a number of RNA and DNA viruses, the antiviral function of MxB has just begun to be revealed.

## Materials and methods

A human CD4+ T cell line SupT1 was utilized to study the replication of an HIV-1 strain named NL4-3. The MxA or MxB cDNA was cloned into a tetracycline-inducible expression vector and stably transduced into SupT1 cells. The endogenous MxB was induced with interferon-a2b. MxB-resistant HIV-1 was selected through growing HIV-1_NL4-3_in MxB-expressing SupT1 cells.

## Results

Ectopic expression of MxB in SupT1 cells, but not MxA, suppressed HIV-1 replication. The inhibition occurred at the integration step. Long-term culture led to the selection of MxB-resistant HIV-1 that carries mutations in viral capsid (CA88) and viral envelope (Env539). A further mutagenesis study revealed that CA88 overcame MxB inhibition; Env539 played a compensatory role by enhancing virus infectivity. Since the CA88 mutation disabled the interaction of HIV-1 capsid with cyclophilin A, a role of cyclophilin A in MxB restriction is hypothesized, which is further supported by the association of MxB with cyclophilin A and the loss of MxB inhibition of HIV-1 as a result of cyclophilin A knockdown or cyclosporine A treatment.

## Conclusions

Taken together, our data suggest that human MxB protein inhibits HIV-1 DNA integration by a cyclophilin A-dependent mechanism.

